# Development of cortical auditory responses to speech in noise in unilaterally deaf adults following cochlear implantation

**DOI:** 10.1371/journal.pone.0239487

**Published:** 2020-09-25

**Authors:** Elsa Legris, John Galvin, Sylvie Roux, Jean-Marie Aoustin, David Bakhos

**Affiliations:** 1 UMR1253, iBrain, Université de Tours, INSERM, Tours, France; 2 Ear Nose and Throat Department, Tours, France; 3 House Ear Institute, Los Angeles, CA, United States of America; Medical University Hannover; Cluster of Excellence Hearing4all, GERMANY

## Abstract

**Background:**

For patients with single-sided deafness (SSD), restoration of binaural function via cochlear implant (CI) has been shown to improve speech understanding in noise. The objective of this study was to investigate changes in behavioral performance and cortical auditory responses following cochlear implantation.

**Design:**

Prospective longitudinal study.

**Setting:**

Tertiary referral center.

**Methods:**

Six adults with SSD were tested before and 12 months post-activation of the CI. Six normal hearing (NH) participants served as experimental controls. Speech understanding in noise was evaluated for various spatial conditions. Cortical auditory evoked potentials were recorded with /ba/ stimuli in quiet and in noise. Global field power and responses at Cz were analyzed.

**Results:**

Speech understanding in noise significantly improved with the CI when speech was presented to the CI ear and noise to the normal ear (p<0.05), but remained poorer than that of NH controls (p<0.05). N1 peak amplitude measure in noise significantly increased after CI activation (p<0.05), but remained lower than that of NH controls (p<0.05) at 12 months. After 12 months of CI experience, cortical responses in noise became more comparable between groups.

**Conclusion:**

Binaural restoration in SSD patients via cochlear implantation improved speech performance noise and cortical responses. While behavioral performance and cortical auditory responses improved, SSD-CI outcomes remained poorer than that of NH controls in most cases, suggesting only partial restoration of binaural hearing.

## Introduction

Speech perception in challenging environments can be difficult even for individuals with normal hearing (NH). Binaural listening allows for some segregation of target speech from competing sounds. Head shadow effects can improve the signal-to-noise ratio (SNR) at one or another ear, depending on spatial locations of the target and masker [1]. Binaural redundancy can offer an advantage with binaural listening compared to monaural listening with either ear alone when speech and maskers are co-located [2–4]. NH listeners may also benefit from binaural squelch, a binaural advantage over monaural listening when the ear with the poorer SNR is added to the ear with the better SNR [5–7]. In cases of single-sided deafness (SSD), binaural cues aren’t available. While SSD patients may benefit from head shadow when the SNR is better in the hearing ear, they are unable to benefit from binaural summation [3, 8, 9]. Given only one hearing ear, SSD patients are unable to use interaural time differences (ITDs) or interaural level differences (ILDs) to extract target information from the background noise [10, 11]. SSD patients also experience impaired sound source localization, and unilateral deafness has been shown to degrade quality of life (QoL) [12–15].

The cochlear implant (CI) offers partial hearing restoration in the deaf ear for SSD patients, and has been shown to significantly improve localization, speech understanding in noise, and QoL, while significantly reducing tinnitus severity [12–14, 16–32]. However, the benefits of cochlear implantation can vary greatly across SSD-CI patients, possibly due to differences in integration of acoustic and electric stimulation patterns. As such, it is unclear how much the CI can truly restore binaural perception. Temporal fine structure (TFS) information (which is important for pitch information, perception of ITDs, etc.) is generally unavailable with CIs [33]. SSD-CI patients have been shown to have poorer ITD sensitivity than do bimodal or bilateral CI patients, suggesting that the CI may not be optimized for combined acoustic and electric hearing in SSD-CI patients [34]. While behavioral measures provide insight into perceptual limits, objective measures such as cortical auditory evoked potentials (CAEPs) may provide further insight into differences in the patterns of activation between acoustic and electric hearing and/or how binaural processes are affected by cochlear implantation.

Previous research has shown that SSD can induce hemispheric asymmetries when monaural auditory stimulation is delivered to the unimpaired ear, as reflected in CAEPs [35], magnetoencephalography (MEG) [36], and functional magnetic resonance imaging (fMRI) [37, 38]. Recent studies have shown evidence of cortical reorganization after cochlear implantation in pediatric and adult SSD-CI patients [39–43]. In a longitudinal CAEP study with SSD-CI patients, Legris et al (2018) observed significant improvements in some cortical responses at mastoid and temporal sites contralateral to the CI ear after 6 months of CI experience, relative to measures before cochlear implantation [42]. In a longitudinal study with SSD-CI children, Polonenko et al (2017) showed that chronic CI stimulation over a six-month period resulted in increased CAEP responses [41]. However, in both these SSD-CI studies, CAEPs were measured using speech stimuli presented in quiet [41, 42].

In adults, CAEPs consist of four peaks: a positive peak (P1) around 50 msec, a negative peak (N1) at approximately 100 msec, followed by another positive peak (P2) at about 200 msec, and a negative peak (N250) around 250ms [44–46]. The presence of these CAEPs components indicates that auditory cortex has been activated and that speech signal has been received [47–49]. The P1 peak is thought to represent early, pre-perceptual processing of acoustic features [50]. The N1 wave is often studied in adults, as it correlated with detection and is thought to reflect principal components of the stimulus [51]. The finer grained properties of the stimulus are reflected by the P2 peak [52]. The N250 peak is thought reflect cortical activity involved in processing of speech stimuli [53]. These CAEP response peaks are influenced by many parameters (e.g., stimulus level). For example, background noise can induce a delay in peak latency and a decrease in peak amplitude [54–57]. In a recent study with 10 young NH participants, Billings (2017) reported that latency was delayed for P1 (+53 ms), N1 (+66 ms) and P2 (+75 ms) peaks for speech in a continuous noise (SNR = -3dB), relative to speech in quiet [58].

Given that one of the primary speech benefits of cochlear implantation for SSD patients is speech understanding in noise, it is unclear how cortical responses might differ between speech presented in quiet or in noise, and how binaural responses to speech in quiet or in noise may be affected by extended CI experience. The objective of this study was to investigate how long-term CI experience (12 months) affects behavioral performance and cortical responses for speech presented quiet or in noise in SSD-CI patients, compare behavioral performance and cortical responses between SSD-CI and NH listeners.

## Methods

### Participants

Six adults (3 men, 3 women), right-handed, French native speakers with acquired SSD participated in this study. SSD-CI patients participated in the study before cochlear implantation and during the first year of CI use. None of the participants had retro-cochlear pathology according to cranial MRI and mini mental state score was 30/30. Table 1 shows SSD-CI patient demographic characteristics. All subjects had profound sensorineural hearing loss (SNHL) in the left ear; the mean unaided air pure tone average (PTA) threshold across 0.5, 1.0, 2.0 and 3.0 kHz was >70 dB HL in the ear to be implanted. Aided disyllable French word recognition (Fournier) was <50% at 60 dB SPL in the ear to be implanted [59]. PTA thresholds were ≤ 20 dB HL in the non-implanted (right) ear. The mean age at implantation was 59±8 years and the mean duration of deafness prior to cochlear implantation was 7.5±9 years. In terms of etiology of deafness, 3 subjects had sudden hearing loss, 1 had Meningitis, and for 2 subjects, the etiology was unknown. Two weeks after surgery, the CI processor was activated. All SSD-CI patients received intensive speech therapy and updated CI processor fittings during the first year of CI use.

**Table 1:** Demographic information for SSD-CI participants.

Group	Subject	Gender	Age at testing (yrs)	Dur deaf (yrs)	Etiology	Right ear PTA (dB HL)	Left ear PTA (dB HL)	CI ear	CI device
SSD	S1	M	53	1.5	Unknown	19	120	L	CI522 ^C^
	S2	F	66	5.5	Sudden	5	83	L	CI512 ^C^
	S3	M	48	2	Sudden	16	120	L	Digisonic SP ^O^
	S4	M	65	2	Sudden	20	95	L	Digisonic SP ^O^
	S5	F	65	20	Unknown	20	120	L	Digisonic SP ^O^
	S6	F	57	6	Meningitis	20	115	L	CI512 ^C^
	Mean ±SD		59 ±8	7.5±9		16.7±6	109±16		
NH	S7	F	61	-	-	17.5	16	-	-
	S8	M	50	-	-	20	20	-	-
	S9	M	51	-	-	5	5	-	-
	S10	F	56	-	-	16	16	-	-
	S11	F	53	-	-	14	13	-	-
	S12	F	53	-	-	8	7	-	-
	Mean ±SD		54±4			13.4 ±5.8	12.8±5.8		

Dur deaf = duration of deafness; PTA = pure-tone average threshold across 0.5, 1.0, 2.0, and 3.0 kHz; ^C^ = Cochlear device; ^o^ = Oticon device.

Six NH adults (2 men, 4 women) served as experimental controls All had PTA thresholds ≤ 20 dB HL and none had any reported neuronal disease. The mean age at testing was 54 ±4 years (range: 50 to 61 years). All NH participants had a mini mental state score of 30/30. Mann-Whitney tests showed no significant differences between the SSD-CI and NH groups in terms of age at testing (p = 0.3, U = 10.5) or gender distribution (p = 0.6, U = 15).

The Ethics Committee of the University Hospital of Tours specifically approved the protocol (N°ID RCB No 2015-A01249-40), and written informed consent was obtained from all subjects by the surgeon before implantation. All patients were recruited from the CI unit of the Otolaryngology Department at University Hospital, Tours, France between 2015 and 2017.

Speech performance in noise and CAEPs in quiet and in noise were measured in SSD-CI participants before cochlear implantation (baseline) and 12 months (12m) after CI activation. Speech performance in noise and CAEPs in quiet and in noise were measured in NH participants in a single test session.

### Speech testing

Sentence recognition in steady, speech-shaped noise was measured using an adaptive procedure. Stimuli consisted of French sentences from the Marginal Benefit from Acoustic Amplification (MBAA) corpus, which consists of 36 lists of 15 sentences [60]. For each condition, a list was randomly selected (without replacement) and sentences within the list were randomly presented (without replacement) in sound field. Three spatial conditions were tested: 1) Speech to the left ear, noise to the right ear (SL-NR), 2) Co-located speech and noise (S0-N0), and 3) Speech to the right ear, noise to the left ear (SR-NL). Note that all SSD-CI participants were implanted in the left ear.

All participants were tested in sound field with binaural listening. After implantation, SSD-CI participants were tested using their clinical processors and settings. Speech was presented at 65 dBA and the noise level was adjusted in 5-dB steps according to the correctness of the response. If the participant repeated the entire sentence correctly, the SNR was reduced by 5 dB; if not, the SNR was increased by 5 dB. The final 6 reversals in SNR were averaged as the speech reception threshold (SRT), defined as the SNR required to produce 50% correct whole sentence recognition.

### Cortical auditory evoked potentials (CAEPs)

#### Stimuli

The speech stimulus used for the cortical recordings was /ba/ produced by a female talker and recorded in a soundproof booth. The fundamental frequency (F0) = 198 Hz, the first formant (F1) = 779 Hz, the second formant (F2) = 1369 Hz, the third formant (F3) = 2720 Hz, and the duration of the stimulus = 125 ms. A total of 1150 stimuli were presented at 70 dBA via 2 loudspeakers situated at 1.3 m away from the subject and -45° and +45° relative to center. Stimuli were presented with a constant inter-stimulus interval of 700 ms (offset to onset); CAEPS were recorded for speech stimuli presented in quiet and in continuous steady white noise at -5 dB SNR; speech and noise were presented from each speaker (i.e., no spatial cues). The -5 dB SNR was used because it corresponded to the mean SRT obtained for S0-N0 before cochlear implantation (-5±4.7 dB). The presentation order for the quiet and noise conditions was randomized across subjects. The CAEP recordings lasted approximately 20 minutes each for the quiet and noise conditions.

#### Electroecephalogram (EEG) data recording

During EEG recording, participants sat on a comfortable armchair in a dimly lit, sound-attenuated room, and watched a silent movie. EEG data were recorded using Compumedics System Neuroscan EEG system (Synamps RT amplifier and Curry 7 software) with 64 electrodes referenced on line to the nose; note that after cochlear implantation, only 61 of the 64 electrodes could be used due to the presence of the CI transmitter coil. All electrodes were placed according to the international 10–20 electrode placement standard. After cochlear implantation, electrodes situated near the CI transmitter coil couldn’t be placed (average number of unplaced electrodes at 12m: 1.8±0.97). Electrode impedances were kept below 5 kΩ. In addition, electrooculogram (EOG) activity was recorded from electrodes placed at the outer canthi of both eyes (horizontal EOG) and above and below the right eye (vertical EOG). The EEG data were recorded with a sampling frequency of 500 Hz and low-pass filtered at 200 Hz. The stimulus presentation was controlled by Neuroscan Stim² software.

EEG analysis was performed using EEGLAB [61] running in the Matlab environment (Mathworks, Natick, MA). First, EEG recordings were filtered by a band-pass filter (0.3–70 Hz). EEG periods recorded during subject movement were identified visually and rejected; the mean artifact rejection was less than 25% per participant for test interval. Extended infomax independent component analysis (ICA) implemented in EEGLAB was applied to the continuous data from each EEG to reduce CI-related artifact, as in Debener et al. (2008) [62]. ICA components representing CI artifacts were identified by the centroid on the side of the implanted device time-locked to the auditory stimulation and had large amplitude. Independent components representing common EEG artifacts (e.g., eye blink and saccade) were visually identified and removed along with those components representing the CI artifacts. The total numbers of components were 64. On average, 4.5 were removed (range: 2 to 8) at interval 12m in quiet and 4.6 (range: 2 to 7) in noise. Afterwards, EEG was segmented into epochs from -100 to 500 ms relative to the stimulus onset. The epochs were baseline-corrected relative to a 100-ms pre-stimulus time window, and a digital zero-phase-shift low-pass filter of 30 Hz was applied, as in our previous related study [42], to preserve waves in the latency range between 50 ms and 1 s and to remove high frequency noise [63, 64]. The mean number of epochs were 748 in quiet and 746 in noise at baseline for the SSD-CI and NH participants, and 692 in quiet and 700 in noise for the SSD-CI participants at 12m.

CAEP analysis was performed with ELAN software [65], and scalp potential maps were created from the CAEP data [66]. Mean averaged waveforms for each eliciting stimulus were obtained separately for each participant. Data from missing electrodes (3.8±2.5) for SSD-CI participants were interpolated. Mean latencies and amplitudes of P1, N1 and P2 were measured at the peak by visual inspection from baseline to peak for each participant, at fronto-central electrode Cz. The global field power (GFP) waveform, which is the standard deviation across channels as a function of time, was used to quantify simultaneous activity from all electrode sites [67]. The GFP waveform presented positive peaks (P1, N1 and P2 waves), which were identified by visual inspection. Peak identification was reviewed by a second investigator to check the consistency of the data with an inter-judge agreement rate of 95%. The two investigators found a consensus for the remaining 5%. Scalp potential maps were generated using a two-dimensional spherical spline interpolation [66] and a radial projection from Cz (top views), with respect to the length of the meridian arcs, across a window of -20 to +20 ms around the P1, N1 and P2 peaks.

#### Statistical analysis

For SSD-CI participants, non-parametric Wilcoxon tests were used to assess the effect of cochlear implantation (baseline pre-activation vs. 12m post-activation) on speech understanding in noise. For CAEPs, a multivariate analysis of covariance (MANCOVA) was performed, with test session (baseline vs 12m), condition (quiet vs. noise) and CAEP components (P1, N1 and P2) as fixed factors, peak amplitude and latency as dependent variables, and participant as the co-varying factor. The significance level was p<0.05 and post-hoc Bonferroni pairwise comparisons were performed for significant effects and/or interactions. Behavioral and electrophysiological data across time were compared using non-parametric Spearman correlations.

For the NH group, a Wilcoxon test was used to compare peak amplitude and latency for P1, N1 and P2 in quiet and in noise. Behavioral and CAEP data were compared between SSD-CI and NH participants using Mann-Whitney tests.

## Results

### Speech testing

**Fig 1:**
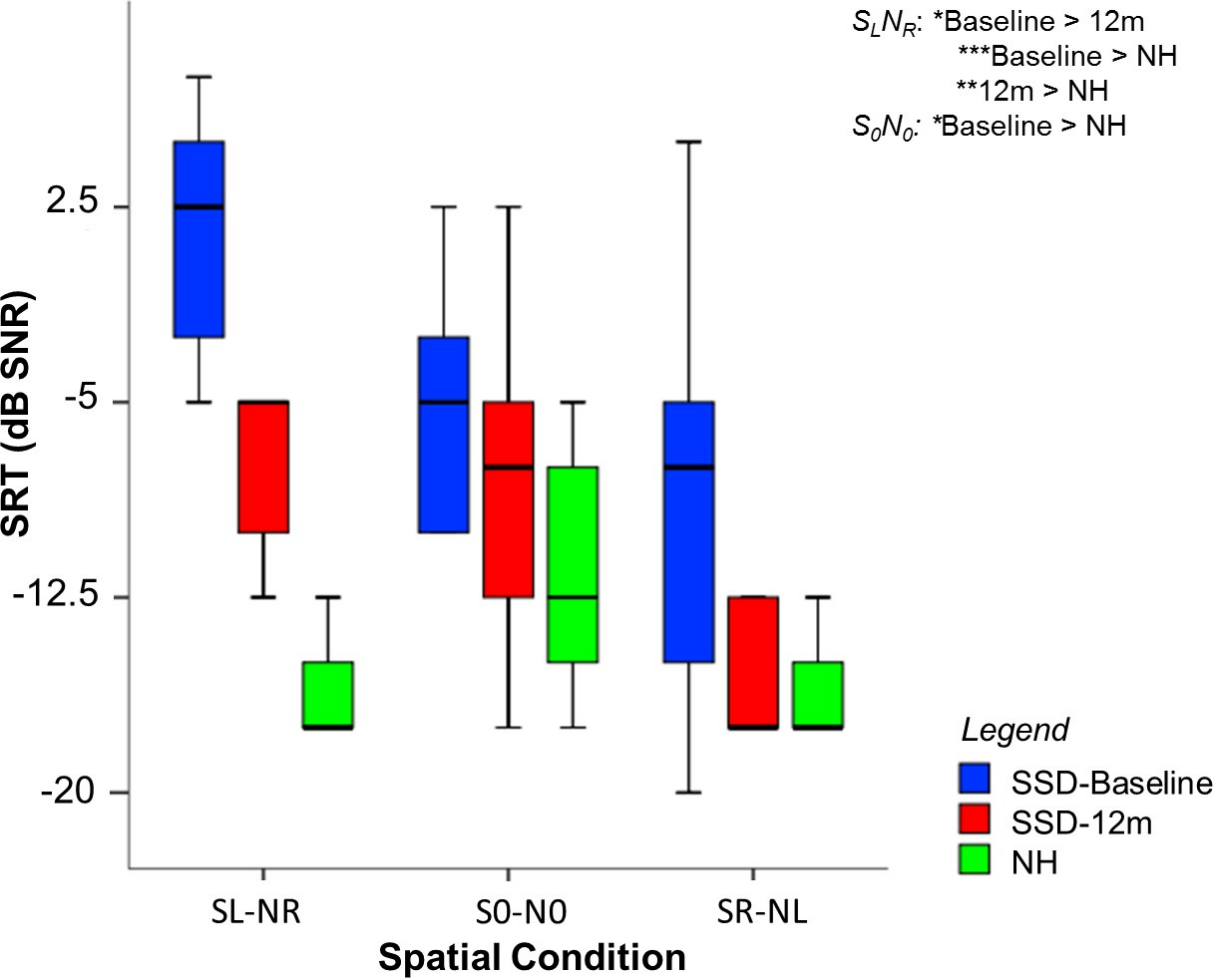
Boxplots of SRTs in noise for the three spatial conditions: SL-NR, S0-N0, and SR-NL. The blue and red boxes show SSD-CI SRTs at baseline at 12m post-activation, respectively; the green boxes show NH data. The boxes show the 25^th^ and 75^th^ percentiles, the error bars show the 5^th^ and 95^th^ percentiles, the solid line shows the median. Significant differences are shown at top right (* = p < 0.05, ** = p < 0.01, *** = p < 0.001, from Wilcoxon tests).

Fig 1 shows boxplots of SRTs in noise for SSD-CI and NH participants for the 3 spatial conditions. For SSD-CI participants, the mean difference in SRTs between baseline and 12m was -7.9±5.7 dB for SL-NR, -2.9±4.2 dB for S0-N0, and -4.2±5 dB for SR-NL. A significant improvement in SRTs at 12m was observed only for SL-NR (p = 0.034, W = -21). For SL-NR, SRTs were significantly better for NH than for SSD-CI listeners at baseline (p = 0.00043, U = 0) and 12m (p = 0.0054, U = 0.5). For S0-N0, performance was significantly better for NH than for SSD-CI listeners at baseline (p = 0.043, U = 5), but not at 12m (p = 0.34, U = 11.5). For SR-NL, there was no significant difference in SRTs between NH and SSD-CI listeners at baseline (p = 0.14, U = 7.5) or 12m: p = 0.85, U = 7.5).

### Cortical auditory responses

**Fig 2:**
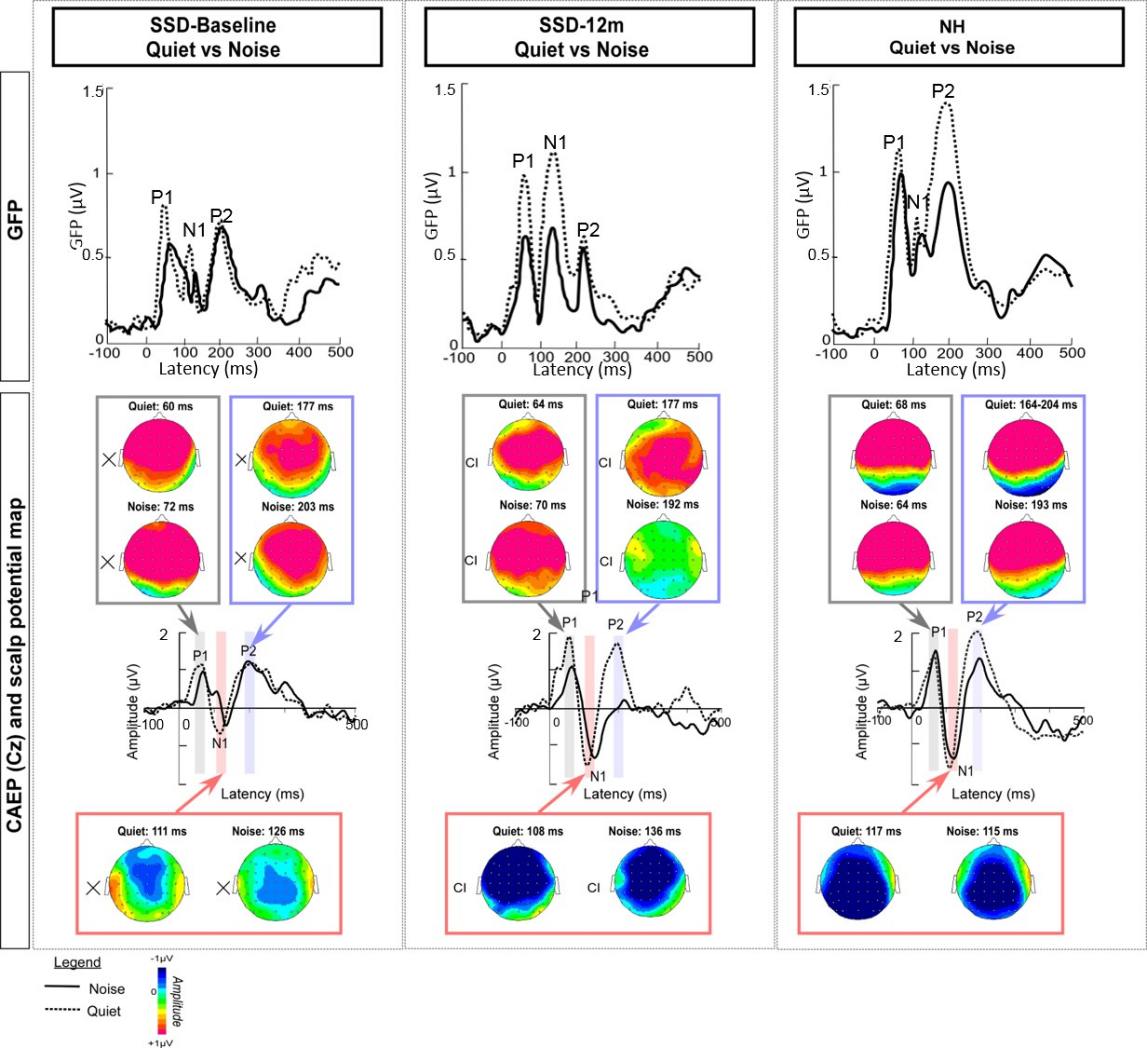
Mean surface recordings for SSD-CI participants at baseline (left panels) and at 12m post-activation (middle panels), and for NH participants (right panels) in quiet (dotted line) and in noise (solid line). The top panels show mean global field power (GFP). The lower panels show CAEPs at Cz, with topographic distributions (top view) of mean average referenced to surface responses at P1, N1 and P2 peak latencies.

Fig 2 shows GFP and CAEPs at Cz at baseline and 12m post-activation for stimuli presented in quiet or in noise. Responses to /ba/ stimuli were maximal over fronto-central electrodes and mainly involved a large negative wave N1 and two large positive waves P1 and P2 culminating at the vertex. Scalp potential maps are shown for each peak (P1, N1 and P2).

#### NH control group

Complete results for Wilcoxon tests comparing responses in quiet and noise for the NH control group are shown in Table 2. For GFP, no significant differences were observed between quiet and noise for P1, N1 and P2 peak amplitude. At Cz, P2 peak amplitude was significantly higher in quiet than in noise (p = 0.036).

**Table 2:** Results of Wilcoxon comparisons between quiet and noise for GFP and Cz responses for the NH control group.

			Quiet (mean±STD)	Noise (mean±STD)	p	W
Amplitude (μV)	GFP	P1	1.25±0.75	1.03±0.56	0.16	-15
		N1	0.82±0.28	0.75±0.25	0.99	1
		P2	1.41±0.65	0.93±0.54	0.09	7
	CZ	P1	1.22±1.38	1.85±1.26	0.75	4
		N1	-1.38±1.96	-1.28±1.37	0.81	3
		P2	2.46±1.24	1.62±0.86	*0*.036*	-21
Latency (ms)	GFP	P1	67±5	78±7	*0*.035*	21
		N1	123±21	133±22	*0*.035*	21
		P2	193±23	212±37	0.17	14
	CZ	P1	57±1	66±8	*0*.034*	21
		N1	105±13	116±14	*0*.031*	21
		P2	196±9	184±13	0.059	19

The asterisks and italics indicate significant differences (p<0.05).

For GFP, P1 latency was significantly longer in noise than in quiet (p = 0.035); N1 latency was also significantly longer in noise (p = 0.035). At Cz, P1 latency was significantly longer in noise than in quiet (p = 0.034); N1 latency was also significantly longer in noise than in quiet (p = 0.031). There was no significant difference between quiet and noise for GFP or at Cz.

Scalp map potential analysis (Fig 2) did not show any clear differences in activity between quiet and noise.

#### SSD-CI subjects

Mean peak amplitudes and latencies for GFP and at Cz for SSD-CI participants are shown at the top of Table 3, and complete results for the MANCOVA analyses are shown at the bottom of Table 3. For GFP, a significant effect of CI experience was observed (p<0.01). In quiet, P1 amplitude was significantly higher at 12m than at baseline (p<0.01). N1 peak amplitude was significantly higher at 12m than at baseline in quiet, and significantly higher at 12m than at baseline in noise (p<0.01). A significantly longer P2 latency was observed at 12m than at baseline in quiet and in noise (p<0.01 in both cases). P1 peak amplitude was significantly higher in quiet than in noise at baseline and at 12m (p<0.001 in both cases). N1 peak amplitude was significantly higher in quiet than in noise at 12m (p<0.001); there was no significant difference between quiet and noise at baseline (p>0.05). No significant differences between quiet and noise were observed for P2 peak amplitude, or for P1, N1, P2 peak latency (p>0.05 in all cases).

**Table 3:** Top: Mean CAEP amplitudes and latencies for GFP and at CZ for SSD-CI participants, in quiet and in noise and at baseline and after 12 months of CI experience. Bottom: Results of MANCOVA tests on GFP and Cz responses for CI experience and listening condition (quiet vs. noise).

					Quiet							Noise	
					Baseline				12m			Baseline	12m
					Mean±STD				Mean±STD			Mean±STD	Mean±STD
GFP		Amplitude (μV)		P1	0.8±0.26				1.09±0.4			0.57±0.14	0.6±0.3
				N1	0.65±026				1.14±0.3			0.48±0.17	0.72±0.16
				P2	0.77±0.12				0.79±0.32			0.72±0.26	0.6±0.14
		Latency (ms)		P1	61±14				57±10			70±13	66±12
				N1	116±14				133±25			112±19	127±24
				P2	191±30				211±16			186±18	220±43
Cz		Amplitude (μV)		P1	1.83±1.32				1.41±0.93			1.12±0.65	1.6±0.86
				N1	-1.27±0.62				-1.67±1.35			-0.64±0.65	-1.46±1.14
				P2	1.23±1.19				1.51±1.62			1.25±0.68	0.37±0.84
		Latency (ms)		P1	66±22				63±13			72±15	71±13
				N1	109±8				108±11			126±15	137±7
				P2	174±18				176±20			203±13	192±26
						dF	F	p		ŋ^2^	Post-hoc (p<0.05)		
GFP	CI experience (baseline, 12m)		Amplitude			1	8.25	*0*.01*		8.25	12m > Baseline: P1 in quiet; N1 in quiet and noise		
			Latency			1	6.67	*0*.01*		6.67	12m>Baseline: P2 in quiet and noise		
	Listening condition (quiet, noise)		Amplitude			1	19.06	*<0*.001*		19.06	Quiet > Noise: P1 at baseline and 12m; N1 at 12m		
			Latency			1	0.24	0.63		0.24			
	CI experience x Listening condition		Amplitude			1	3.16	0.08		3.16			
			Latency			1	0.2	0.66		0.20			
Cz	CI experience (baseline, 12m)		Amplitude			1	1.04	0.31		1.04			
			Latency			1	0.02	0.89		0.02			
	Listening condition (quiet, noise)		Amplitude			1	0.81	0.37		0.81			
			Latency			1	20.29	*<0*.001*		20.29	Noise > quiet: N1 and P2 baseline, 12m		
	CI experience x Listening condition		Amplitude			1	1.11	0.3		1.11			
			Latency			1	0.09	0.77		0.09			

The asterisks and italics indicate significant differences. Bonferroni corrected post-hoc comparisons are shown in the right-most column.

At Cz site, there was no effect of CI experience on peak latencies (p>0.05); however, significant differences were observed between quiet and noise. N1 and P2 latencies were significantly shorter in quiet than in noise at baseline and 12m (p<0.001 in all cases). For peak amplitudes, no significant effects were observed for CI experience or between quiet and noise.

Scalp potential map (Fig 2) showed similar activity in quiet and in noise for P1 at baseline and 12m. Similar activity in quiet and in noise was also observed for P2 wave at baseline, but the positive frontocentral field activity was lower in noise than in quiet at 12m. For N1, the negative field at frontocentral areas was lower in noise than in quiet at baseline.

#### Comparison between NH control group and SSD-CI participants

**Fig 3:**
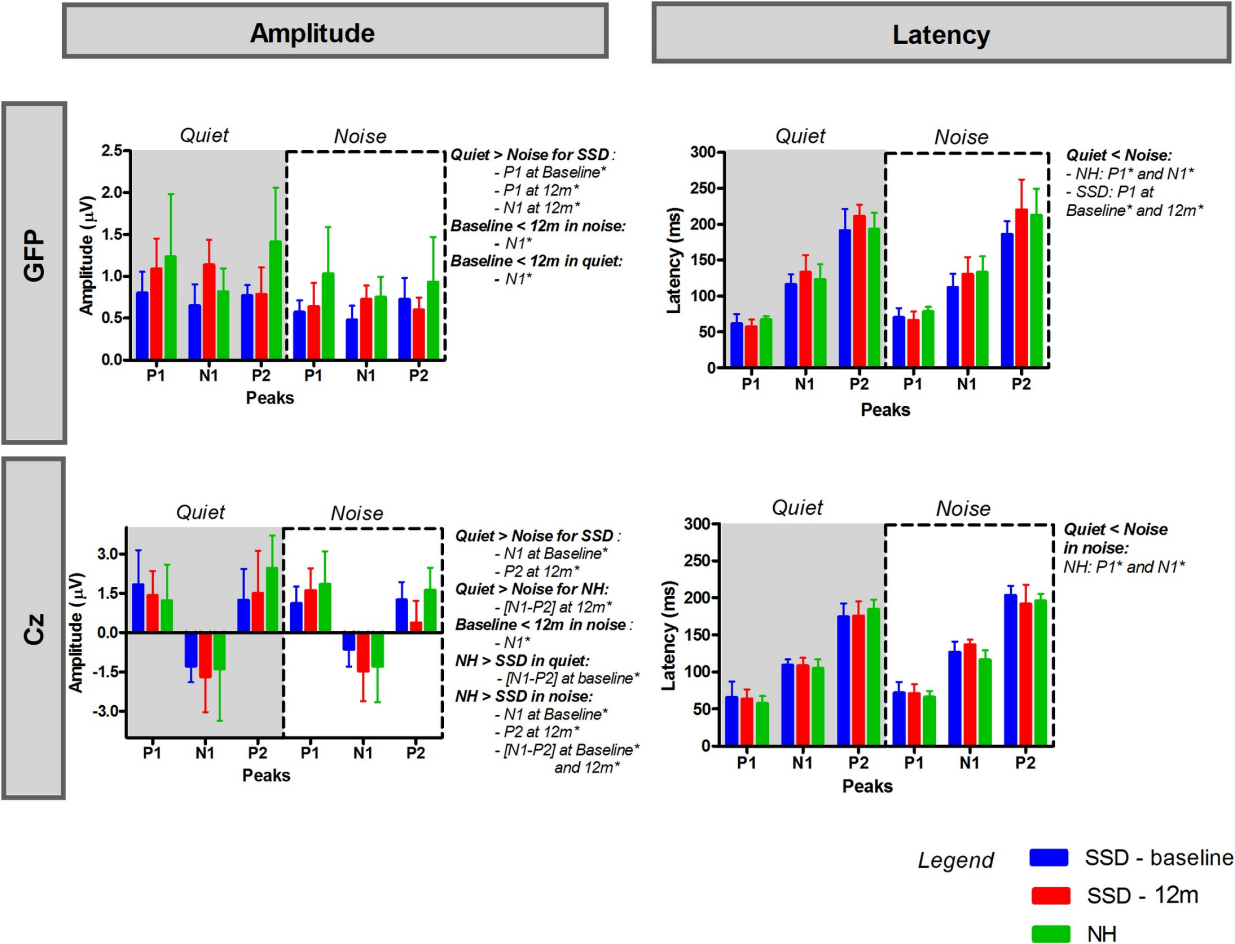
Mean peak amplitude and latency for GFP (top panels) and at Cz (bottom panels) in quiet (grey panels) and in noise (white panels) for SSD-CI participants at baseline (blue) and 12m post-activation (red) and for the NH control group (green). The error bars show the standard deviation.

Fig 3 shows mean GFP and Cz peak amplitudes and latencies in quiet and in noise for SSD-CI participants at baseline and 12m post-activation, and for the NH control group. Complete results for Mann-Whitney tests comparing responses between the SSD-CI and NH participants are shown in Table 4. Significant differences between baseline and 12m were observed for SSD-CI participants, between quiet and noise, and between NH and SSD-CI participants are shown to the right of the panels (* = p<0.05).

**Table 4:** Results of Mann-Whitney comparisons between the NH control group and SSD-CI participants at baseline or 12m for GFP and Cz responses in quiet and in noise.

				Amplitude		Latency	
				p	U	p	U
Quiet	GFP	P1	Baseline	0.380	12	0.570	14
			12m	0.940	17	0.150	8.5
		N1	Baseline	0.240	10	0.870	7.5
			12m	0.180	9	0.690	15
		P2	Baseline	0.092	7	0.810	16
			12m	0.130	8	0.130	8
	Cz	P1	Baseline	0.999	18	0.520	13.5
			12m	0.999	18	0.630	14.5
		N1	Baseline	0.290	11	0.870	16.5
			12m	0.590	14	0.870	16.5
		P2	Baseline	0.310	11	0.230	10
			12m	0.260	10.5	0.570	14
Noise	GFP	P1	Baseline	0.240	10	0.260	10.5
			12m	0.310	11	0.092	7
		N1	Baseline	0.065	6	0.220	10
			12m	0.940	17	0.810	16
		P2	Baseline	0.630	14.5	0.260	10.5
			12m	0.470	13	0.570	14
	Cz	P1	Baseline	0.310	11	0.807	16
			12m	0.818	16	0.468	13
		N1	Baseline	*0*.0152*	*3*	0.225	10
			12m	0.818	16	0.070	7.5
		P2	Baseline	0.485	13	0.228	10
			12m	*0*.041*	*5*	0.873	16.5

The asterisks and italics indicate significant differences.

For GFP, there were no significant differences between the NH and SSD-CI groups at baseline or 12m in terms of P1, N1, or P2 amplitude or latency in quiet or in noise (p>0.05 in all cases). At Cz in noise, some significant differences were observed between the NH and SSD-CI groups. The NH control group exhibited significantly lower N1 amplitude (-1.8±0.4 μV) than did SSD-CI participants at baseline (-0.64±0.65 μV) (p = 0.0152, W = 3). P2 amplitude was significantly higher for the NH control group (1.53±0.83 μV) than for SSD-CI participants at 12m (0.37±0.84 μV) (p = 0.041, U = 5). There were no significant differences observed between NH and SSD-CI participants for P1, N1, or P2 peak latencies (p>0.05 in all cases).

The scalp map potential in Fig 2 showed that P1 activity was similar between the NH and SSD-CI participants in quiet and in noise. While P2 activity in quiet was similar between the NH and SSD-CI participants, P2 activity in noise was markedly higher for the NH group than for SSD-CI group at 12m. In noise, the negative field at frontocentral areas for the N1 peak was lower for SSD-CI participants at baseline than for the NH group; at 12m, activity was more comparable between NH and SSD-CI participants.

#### Correlational analyses

Changes in SRTs from baseline to 12m for the different spatial conditions were compared to changes in cortical responses from baseline to 12 m in the SSD-CI participants; complete results for Spearman correlations are shown in Table 5.

**Table 5:** Results of Spearman correlations comparing the change in SRTs (from baseline to 12m) for the different spatial conditions and changes in GFP and CAEP amplitudes and latencies in noise (from baseline to 12m) in SSD-CI participants.

				Change in SRT		
				SL-NR	S0-N0	SR-NL
GFP	Change in amplitude	P1	r	0.33	0.79	0.12
			p	0.50	0.06	0.80
		N1	r	0.64	0.65	0.43
			p	0.18	0.18	0.42
		P2	r	-0.52	0.09	-0.70
			p	0.30	0.92	0.14
	Change in latency	P1	r	-0.03	0.24	0.60
			p	0.99	0.66	0.24
		N1	r	-0.15	-0.62	-0.35
			p	0.80	0.18	0.50
		P2	r	-0.03	-0.74	-0.29
			p	0.99	0.10	0.56
Cz	Change in amplitude	P1	r	-0.15	0.56	-0.46
			p	0.80	0.24	0.35
		N1	r	-0.15	0.65	-0.23
			p	0.80	0.18	0.66
		P2	r	-0.39	0.29	-0.55
			p	0.42	0.56	0.30
	Change in latency	P1	r	-0.68	-0.47	-0.28
			p	0.14	0.36	0.56
		N1	r	-0.58	-0.18	-0.49
			p	0.24	0.71	0.36
		P2	r	-0.70	-0.79	0.29
			p	0.14	0.058	0.56

For GFP, moderate correlations (r≥0.50) were observed between the change in N1 and P2 amplitude and the change in SRTs for SL-NR, and between the change in N1 amplitude and the change in SRTs for S0-N0. Strong correlations (r≥0.70) were observed between the change in P1 amplitude and the change in SRTs for S0-N0, and between the change in P2 amplitude and the change in SRTs for SR-NL. A moderate correlation was observed between the change in N1 latency and the change in SRTs for S0-N0, and a strong correlation was observed between the change in P2 latency and the change in SRTs for S0-N0. However, none of these moderate or strong correlations were significant (p>0.05), most likely due to the limited number of participants (n = 6).

At Cz, moderate correlations were observed between the change in SRTs for S0-N0 and the changes in P1 and N1 peaks amplitudes; a moderate correlation was also observed between the change in SRTs for SR-NL and the change in P2 amplitude. Moderate correlations were observed between the change in SRTs for SL-NR and N1 latency; and a strong correlation was observed between the change in SRTs for SL-NR and P1 and P2 latency, and between the change in SRTs for S0-N0 and P2 latency. Again, none of these correlations were significant (p>0.05), most likely due to the limited number of participants (n = 6).

## Discussion

Consistent with many previous studies [14, 27–30, 42], cochlear implantation improved speech perception in noise, especially when speech was presented to the CI ear and noise to the NH ear. For the present SSD-CI participants, cortical responses N1, and P2 were delayed when speech was presented in noise, relative to presentation in quiet at baseline and 12m (Cz site); P1 and N1 amplitudes were also greater in quiet than in noise (GFP). After 12 months of CI experience, cortical responses changed and became closer to those for the NH control group, suggesting some cortical reorganization. However, behavioral performance and electrophysiological responses remained poorer in SSD-CI patients compared to the NH control group even 12 months of CI experience.

### Development of behavioral and electrophysiological measures after cochlear implantation in SSD participants

Speech performance was poorer when for the SL-NR than for the SR-NL spatial condition for SSD-CI patients. These observations are consistent with aural preference studies in animals and adult humans [68, 69]. Due to the constant advantage of the stronger ear (the right ear in the present SSD-CI patients), it is an inevitable consequence that higher order areas of the brain (including the linguistic network) preferentially process the input from the stronger ear [68, 69]. SSD induces a reduced binaural suppression in the cortex ipsilateral to the hearing ear [70], which results in an overrepresentation of the hearing ear (“aural preference syndrome”) [68]. After cochlear implantation, a significant improvement in SRTs was observed only for the SL-NR condition, consistent with head shadow benefits observed in previous studies [12, 16, 22, 23, 29, 30, 32]. Some previous studies have also reported significant improvements for the S0-N0 and SR-NL spatial conditions after cochlear implantation [12, 22] possibly due to procedural learning or some unclear benefit of the CI on performance (e.g., tinnitus reduction, reduced cognitive load). In this study, no significant improvements were observed for the S0-N0 and SR-NL conditions, possibly due to the limited number of participants.

Cortical development for SSD-CI participants was compared between baseline and after 12 months of CI experience. A significant change in N1 peak amplitude at frontocentral sites (Fig 2) was observed for CAEPs and GFP for speech in noise, and a significant change was also observed for GFP for speech in quiet. Scalp potential maps for speech in noise also revealed a reinforcement of the negative field at frontocentral areas after 12 months of CI experience (Fig 2). This underlines the activation of generators situated at the supratemporal auditory cortex in noisy environments after cochlear implantation [71, 72]. Similar improvements in CAEPs after extensive CI experience have been observed for pediatric SSD-CI patients in quiet [39, 41] and noise [40], and for adult SSD-CI patients in quiet [42, 43]. The CI appears to restore temporal, spectral and spatial auditory cues necessary for binaural integration [73, 74]. Previous studies have shown significant correlations between CAEP amplitudes (especially N1 peaks) with speech perception in noise [75–78]. In the present study, no significant correlations were observed between changes in cortical responses and changes in behavioral performance (Table 5) after 12 months of CI experience, most likely due to the limited number of participants. However, a number of moderate (r≥0.50) and strong (r≥0.70) correlations were observed, suggesting that improvements in behavioral performance may have been associated with changes in cortical responses.

Note that the SSD-CI participants reported that they used their device every day, and all received extensive auditory training and fitting adjustments during the first year post-activation, which may have contributed to the better speech performance after receiving the implant. Indeed, auditory training has been shown to improve CAEPs in adult CI patients [79, 80].

### Effect of noise on cortical responses

The restoration of binaural function with the CI appeared to reduce the gap between cortical responses measured in quiet and in noise. This could suggest better auditory processing in noise at cortical level after cochlear implantation. As shown in Fig 2, P1 and N1 peak amplitudes were clearly higher and latencies shorter in quiet than in noise for GFP and CAEP at baseline and 12m. Statistical analyses (Tables 2 and 3) showed significant differences in cortical responses between quiet and noise for SSD-CI participants (P1 and N1 peak amplitude for GFP), and for the NH control group (P2 peak amplitude at Cz). Similarly, significant delays for latency were observed in noise for SSD-CI participants (N1 and P2 latencies at Cz) and for the NH control group (P1 and N1 latencies for GFP and at Cz). The pattern of results was consistent with previous studies showing poorer morphology of CAEPs as the SNR became was reduced [54–57]. In addition, scalp map potential at baseline for SSD-CI patients showed a greater negative field at frontocentral site in quiet than in noise for N1 (Fig 2). This difference between quiet and noise was reduced after 12 months of CI experience. These results underline the importance of restoring binaural hearing for SSD patients, in order to reduce difficulties in speech perception in noisy environments. Kral et al. [81] showed that the success of auditory restoration depended on the age at implantation, highlighting the importance early intervention via CI for SSD patients.

### SSD-CI participants versus NH control group

While speech understanding in noise generally improved after cochlear implantation, SRTs remained poorer for SSD-CI participants than for the NH control group, consistent with previous studies [12, 16, 23]. For speech in quiet, CAEP amplitudes and latencies were similar between SSD-CI and NH participants. For speech in noise, CAEPs were markedly different between SSD-CI and NH participants. At baseline, N1 peak amplitude was lower in SSD-CI participants than in the NH group. After 12 months of CI experience, N1 wave activity became more comparable between SSD-CI and NH participants (as shown in scalp map potential; Fig 2). This may suggest modifications to the mesencephalic reticular activating system in noise with extended CI experience [51, 82, 83]. However, P2 amplitude remained lower for SSD-CI participants than for the NH group, even after 12 months of CI experience. Taken together, the behavioral and electrophysiological data suggest that cochlear implantation may only partially restore binaural function in SSD patients.

### Study limits

For CAEP recordings, stimuli were delivered in sound field from two speakers positioned ±45 degrees from center, and recordings were made only for binaural listening (i.e., both ears received sound, before and after cochlear implantation). Monaural stimulation delivered directly to the NH ear via insert earphone and to the CI ear via direct audio input (DAI) may have allowed for greater specificity for cortical responses with acoustic and electric hearing. Also, recordings were not made after cochlear implantation with the CI off, which would have been a good control for baseline recordings, or may have shown possible enhancement to NH responses after cochlear implantation. In the present study, speech stimuli were presented in quiet and in noise to better represent everyday listening conditions [54, 84]. Recording CAEPs in noise also allowed for a more direct comparison to behaviorally measured speech understanding in noise. However, the short /ba/ stimulus used for cortical recordings may not fully reflect ongoing cortical processes involved in understanding sentences in noise. This study also did not explore CI-related issues that might limit binaural perception and/or cortical responses. For example, tonotopic mismatch across ears has been shown to limit binaural integration [85, 86]. The relative loudness of acoustic and electric hearing (which is affected by the amplitude mapping function in CIs) may also affect cortical recordings. Such CI-related issues may limit SSD-CI users’ integration of acoustic and electric hearing, and may explain poorer speech performance and/or lower amplitudes for CAEPs in noise, relative to NH listeners. It would also be interesting to measure cortical responses using the spatial configurations used for behavioral testing (SL-NR, S0-N0, SR-NL), where CAEPs might differ across spatial configurations and be better correlated with behavioral data.

Another limit of the present study is the small number of SSD-CI participants (n = 6). In France, there are limited numbers of SSD patients that received a CI as part of a research protocol [87]. While the number of SSD-CI participants is small in the present study, it is larger than many other SSD-CI cortical recording studies, where the number of patients is often 3 or less [39, 40, 43]. Also, only SSD-CI patients implanted in the left ear participated in the study; as such, laterality effects were not explored. The side of deafness has been shown impact cortical reorganization [38, 88]. SSD patients implanted in the left ear were recruited for the present study to decrease heterogeneity and/or side bias for cortical recordings. All of the present participants had post-lingual onset of SSD. Previous studies have shown greater plasticity for congenital hearing loss [89, 90]. Further studies with a larger cohort are needed to explore effects of the side of deafness and/or duration of deafness on behavioral and electrophysiological measures.

Finally, CI artifact can have a huge impact on CAEP responses. ICA (as used in this study), while efficient in removing CI artifact, is not a truly objective approach and might also remove part of the signal [91]. It is unclear how the responses might be affected by ICA, compared to other artifact removal methods [92]. We tried to minimize potential negative effects of ICA on electrophysiological data by having two investigators review responses.

## Conclusion

Improvements in speech understanding and noise and changes in cortical auditory responses were observed in SSD-CI participants after 12 months of experience with their CI. While behavioral performance remained poorer than that of the NH control group, cortical responses for SSD-CI participants became more similar to those of the NH group after 12 months of CI experience. Differences is cortical responses were observed for speech presented in quiet or in noise for both subject groups. The data suggest that cochlear implantation may restore some binaural function in SSD patients, and that some cortical reorganization may occur as patients gain experience with their device.

## Supporting information

S1 Data (XLSX)
